# Discovery and Validation of a SIT1-Related Prognostic Signature Associated with Immune Infiltration in Cutaneous Melanoma

**DOI:** 10.3390/jpm13010013

**Published:** 2022-12-21

**Authors:** Ming Jia, Chengfei Liu, Yuean Liu, Zhengqiang Bao, Yuhua Jiang, Xifeng Sun

**Affiliations:** 1 Department of Cancer Center, The Secondary Hospital, Cheeloo College of Medicine, Shandong University, Jinan 250033, China; 2 Department of Pharmacy, Shandong Cancer Hospital and Institute, Shandong First Medical University and Shandong Academy of Medical Sciences, Jinan 250117, China; 3 Department of Emergency Medicine, Shandong Provincial Clinical Research Center for Emergency and Critical Care Medicine, Institute of Emergency and Critical Care Medicine of Shandong University, Qilu Hospital of Shandong University, Jinan 250012, China

**Keywords:** gene signature, *SIT1*, immune response, skin cutaneous melanoma, tumor mutation burden, nomogram

## Abstract

Signaling threshold regulating transmembrane adaptor 1 (*SIT1*) encodes a disulfide-linked homodimeric lymphocyte-specific glycoprotein involved in immune cell activation. However, the relationship between *SIT1* and the prognosis of skin cutaneous melanoma (SKCM) and tumor-infiltrating lymphocytes remains elusive. Here, we first compared the differences in SIT1 expression levels between SKCM tissues and adjacent normal tissues. Next, we found that the immune cell infiltration levels and signature pattern of immune infiltration were positively associated with the *SIT1* gene mRNA levels. TCGA_SKCM RNA-seq data unveiled that the *SIT1* upregulated several immune-associated signaling pathways in GSEA analysis. The high expression of *SIT1* was closely related to improved survival in patients with SKCM. A pathway enrichment analysis of *SIT1*-associated immunomodulators indicated the involvement of the NF-κB signaling pathways. Based on SIT1-associated immunomodulators, we built a 13-gene signature by LASSO Cox regression which served as an independent prognostic factor for the survival of melanoma patients. By using the signature risk score, we achieved a good prediction result for the immunotherapy response and survival of SKCM patients. Our findings provided evidence for *SIT1*’s implication in tumor immunity and survival of SKCM patients. The nominated immune signature is a promising predictive model for prognosis and immunotherapy sensitivity in SKCM patients.

## 1. Introduction

Skin cutaneous melanoma (SKCM) with an aggressive phenotype is one of the most commonly diagnosed and has the highest mortality rate among all types of skin cancers, which causes approximately 57,000 deaths worldwide each year [1]. Melanoma was reported to exhibit high immunogenicity and immune cell infiltration in previous studies [2,3]. Over the past decade, immune therapies have dramatically changed the landscape of SKCM treatment [4,5,6,7]. Pembrolizumab and nivolumab have been approved for the treatment of patients with advanced or metastatic melanoma and as an adjuvant treatment for patients with a high risk of relapse in many areas across the world [8,9].

However, not all SKCM patients would benefit from immunotherapy. In fact, only a small percentage of melanoma patients showed good responses and improved long-term survival when receiving immunotherapy. The molecular mechanisms of immunotherapy resistance are complex, whereas tumor stroma-intrinsic factors such as insufficient tumor antigenicity, tumor-intrinsic interferon-γ signaling repression, tumor loss of MHC, oncogenic signaling hyperactivation, loss of tumor suppressor, tumor dedifferentiation, and stemness play important roles in the process [10]. Immune markers such as PD-L1 expression and levels of immune cell infiltration, such as CD8 + T cell infiltration, may help in identifying the patients with good responses to immunotherapies [11,12], however, there were exceptions in many SKCM cohorts as well, which may be due to local immunosuppressive factors and T cell dislocation [13,14]. So far, no ideal markers have been found for SKCM patients’ sensitivity to immunotherapy. Systemic knowledge of factors affecting effective immunotherapies for melanoma is still lacking [15]. Thus, the molecular features representing the complex intratumoral immune microenvironment still need investigation. Therefore, it is essential to fully understand SKCM immunology and the underlying mechanisms to improve the success rate of immunotherapy. With the appearance of high-dimensional datasets and advanced bioinformatics algorithms [16,17], it is realistic to further investigate the immune activity and multiple-gene expression in multiple tumor types, which facilitate us to study the molecular characteristics affecting immune cell infiltration, response to immunotherapy, and the prognosis of SKCM patients.

Signaling threshold regulating transmembrane adaptor 1 (SIT1), encoded by the *SIT1* gene, represents a disulfide-linked homodimeric glycoprotein belonging to the lymphocyte-specific transmembrane adaptor protein family, which is a group of molecules that affect the immunity processes [18]. SIT contains five tyrosine-based signaling motifs in the cytoplasmic domain that could mediate the binding affinity of the SH2 domain with intracellular signaling molecules. Previous studies have shown that SIT inhibits TCR-mediated signaling [19,20]. However, the mechanism is not clear. Since SKCM has shown a good response to cancer immunotherapy and the immune implication of the *SIT1* gene in SKCM remains mostly unknown so far, we systematically elucidate the association between *SIT1* and SKCM immunity as well as the *SIT1*-mediated immune response-associated signaling pathways. Association between *SIT1* gene expression and SKCM patients’ survival has also been discussed. Then, we generated a prognostic immune signature using *SIT1*-associated immunomodulators. Melanoma patients were divided into two groups according to the median of signature-based risk scores in order to study the difference in tumor mutation burden (TMB) and the efficacy of immunotherapy. Finally, we built a nomogram by combining the signature-based risk score with other important clinical features based on the TCGA database, followed by validation in an independent GEO cohort. In summary, our present work may promote the illustration of immune cell infiltration and prognostic factors in SKCM patients.

## 2. Materials and Methods

### 2.1. Data Preparation

Detailed information on mRNA expression (HTSeq—FPKM, *n* = 472), phenotype (*n* = 481), and survival (*n* = 479) from the Cancer Genome Atlas (TCGA_SKCM) database was downloaded from the GDC hub of UCSC Xena website (http://xena.ucsc.edu/public) on 15 April 2022. Tumor samples from the metastatic lesion were discarded from the present study for patients for whom two or more samples have been denoted. Normalized mRNA expression data of fragments per kilobase per million were converted to transcripts per million (TPM) and log-transformed (log2(TPM + 1)) before data analysis. Ensemble IDs were transferred into gene symbols according to the gene probe map downloaded from the GDC hub. A total of 450 tumor samples with complete overall survival data in the TCGA_SKCM dataset were selected for further study after the data filtering process.

Through the *GEOquery* package in R version 4.0.5 (R Foundation for Statistical Computing, Vienna, Austria) [21], we downloaded the normalized mRNA expression data of five skin melanoma GEO datasets (GSE65904, GSE22153, GSE19234, GSE98394, and GSE35640) from the Gene Expression Omnibus (GEO) database. We conducted signal intensity normalization across arrays of the above datasets by using the normalizeBetweenArrays function from the *limma* package in R software. The gene symbols were converted from the probe map according to the previous study [22].

We downloaded the simple nucleotide variation data (workflow type: VarScan2 Variant Aggregation and Masking) from the GDC database (https://portal.gdc.cancer.gov/) (accessed on 15 April 2022) of the TCGA_SKCM cohort. The maf file was analyzed by the *maftools* package in R.

The differences in *SIT1* expression between tumorous and normal tissues in various cancer types were analyzed in the Oncomine database (https://www.oncomine.org/resource/main.html) (accessed on 15 April 2022) [23]. The cut-off of *p* values and fold change were 0.001 and 1.5, respectively. All methods were carried out in accordance with relevant guidelines and regulations.

### 2.2. Correlation between SIT1 and Tumor Immune Cell Infiltration

*SIT1* expression and its association with immune cell infiltration in SKCM and UVM (uveal melanoma) were analyzed through the TIMER website (https://cistrome.shinyapps.io/timer/) (accessed on 15 April 2022). The correlation between tumor purity and gene expression levels was shown in the left-most panel [24]. Besides, the interconnections between *SIT1* mRNA levels and expression of immune cell biomarkers were analyzed through TIMER and Gene Expression Profiling Interactive Analysis (GEPIA) websites [25] using correlation modules. Immune cell biomarkers were selected from previous studies [26,27,28]. Spearman’s correlation was used to investigate the association between *SIT1* and immune cell biomarkers, and results with *p* < 0.001 were considered significant.

*SIT1*-associated signaling pathways were studied by gene set enrichment analysis (GSEA) [29] using the GSEA software version 4.0.0 (UC San Diego and Broad Institute, California, USA), which was downloaded from the website (software.broadinstitue.org/gsea/index.jsp) (accessed on 15 April 2022). *C2.cp.kegg.v7.4. symbols.gmt* was chosen as the gene set database. Patients from TCGA_SKCM datasets were divided into two groups (*SIT1*_high_ vs. *SIT1*_low_) according to the median of the *SIT1* mRNA expression data. The pathways were determined following *p*_FWER_ < 0.05 and normalized enrichment score (NES) > 1.

### 2.3. Immunomodulators

The TISIDB (http://cis.hku.hk/TISIDB/) (accessed on 15 April 2022) [30] database was used to screen the immunomodulators associated with *SIT1* expression. The inclusion criteria were that immunoinhibitors or immunostimulators must have a *p*-value < 0.05 when correlated with *SIT1* gene expression by the Spearman correlation test.

The top 50 immunomodulators associated with the *SIT1* gene were selected by the Comparison module of the cBioPortal for Cancer Genomics (www.cbioportal.org) (accessed on 15 April 2022). GO annotation and Kyoto Encyclopedia of Genes and Genomes pathway enrichment analysis were carried out using web-based tools (https://string-db.org/) (accessed on 17 April 2022) and WEB-based GEne SeT AnaLysis Toolkit (http://www.webgestalt.org/) (accessed on 17 April 2022) [31] through subjecting the selected genes to the website.

### 2.4. Construction of the Prognostic Model

Gene signatures were generated by putting *SIT1*-associated immunomodulators into the least absolute shrinkage and selection operator (LASSO) Cox regression model. The glmnet package in R was used to complete the regression process. After the genes were selected, a multivariate Cox analysis was used to calculate the corresponding coefficients. The signature scores were calculated by the sum of products of each gene and its corresponding coefficients as follows: score = (CD80 × −0.23723) + (ICOSLG × −0.08386) + (IL2RA × −0.04791) + (KLRK1 × −0.24327) + (TMIGD2 × 0.42982) + (TNFRSF14 × 0.08690) + (TNFRSF4 × −0.12586) + (TNFSF15 × 0.19597) + (TNFSF4 × −0.03398) + (ADORA2A × −0.63896) + (CD274 × −0.08743) + (IDO1 × −0.10688) + (NECTIN2 × 0.15422). To normalize signature scores across different datasets, we transformed the score into a risk score with the following formula: risk score = (score-Min)/absolute (Max). The prognostic accuracy of the risk scores in different datasets was determined using the time-dependent receiver operating characteristic (ROC) curves through the *timeROC* package in R [32].

### 2.5. Construction of Nomogram

A nomogram built through the *rms* package in R software was used to predict a patient’s prognosis. Important characteristics and signature risk scores were included in the nomogram. The patients were stratified into low-, medium-, and high-risk groups by the superior and inferior quartiles of the risk score. The concordance index (C-index) and a calibration curve were used to estimate the predictive accuracy of the nomogram.

### 2.6. Statistical Analysis

Most statistical analyses were conducted using R version 4.0.5 (R Foundation for Statistical Computing, Vienna, Austria). Heatmap plots, violin plots, survival curves, and risk factor analyses were performed in Hiplot (https://hiplot.com.cn) (accessed on 20 April 2022), a comprehensive web platform for scientific data visualization. Wilcoxon tests were conducted to compare gene expression between groups. Generally, results with *p* < 0.05 were considered statistically significant.

## 3. Results

### 3.1. mRNA Level of SIT1 in Various Tumor Types

**Figure 1:**
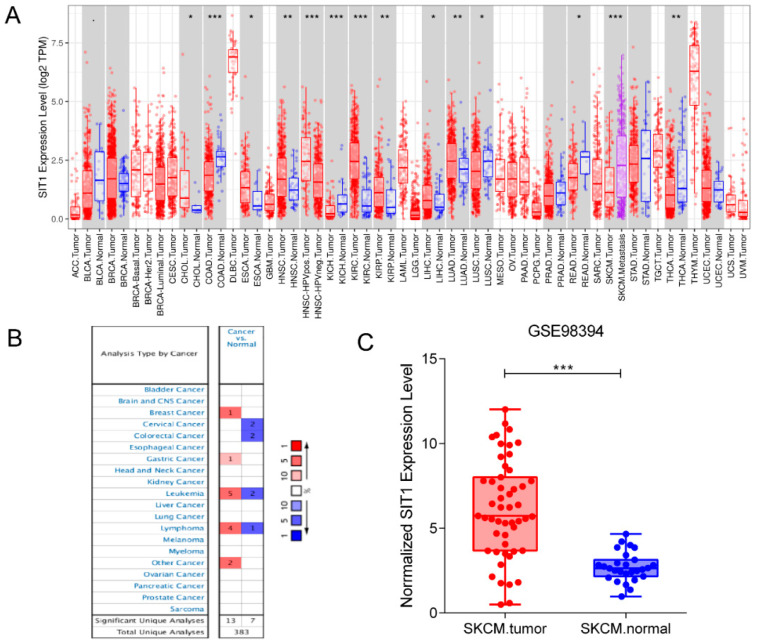
*SIT1* mRNA expression levels in various malignant tumors. (**A**) The mRNA expression levels of *SIT1* between tumor and normal tissues in different tumor datasets from the Oncomine database. (**B**) The mRNA expression levels of *SIT1* between tumor and normal tissues in different tumor datasets from the TCGA database. (**C**) The mRNA expression levels of *SIT1* between tumor and normal tissues in the GSE98394 database. * *p* < 0.05; ** *p* < 0.01; *** *p* < 0.001.

Using the TCGA database, we searched Transcriptome-seq data of *SIT1* in different tumors together with their adjacent normal tissues. *SIT1* expression was higher compared to adjacent normal tissues in cholangiocarcinoma, lung adenocarcinoma, esophageal carcinoma, liver hepatocellular carcinoma, head and neck squamous cell, kidney renal papillary cell carcinoma, and kidney renal clear cell carcinoma. On the contrary, *SIT1* expression was lower in tumor tissues than normal tissues in colon adenocarcinoma, kidney chromophobe, lung squamous cell carcinoma, rectum adenocarcinoma, and thyroid carcinoma (Figure 1A). For skin cutaneous melanoma (SKCM), *SIT1* mRNA was much higher in metastatic tumors than in primary tumors, however, there was no comparison of data for *SIT1* expression in SKCM tissues and adjacent normal tissues because of a very small normal tissue sample size in the dataset.

The Oncomine database was also used to evaluate *SIT1* expression in various human tumors (Figure 1B). The *SIT1* expression levels in breast and gastric cancers were higher than in the adjacent normal tissues (Figure 1B). In contrast, *SIT1* expression in cervical and colorectal cancers was lower compared to the normal tissues in some datasets. In leukemia and lymphoma, the results from different datasets were inconsistent. For SKCM, no significant difference was found between the tumor and normal tissues.

Since the TCGA dataset did not contain a sufficient number of adjacent normal skin tissues, to further evaluate the difference in *SIT1* mRNA expression between the tumor and adjacent normal tissues, we analyzed the difference in *SIT1* mRNA expression in the GSE98394 dataset (Figure 1C). Interestingly, the result showed that *SIT1* expression in tumors was higher compared to adjacent normal tissues; the difference was significant (*p* < 0.001).

### 3.2. Association between SIT1 and Immune Cells

**Figure 2:**
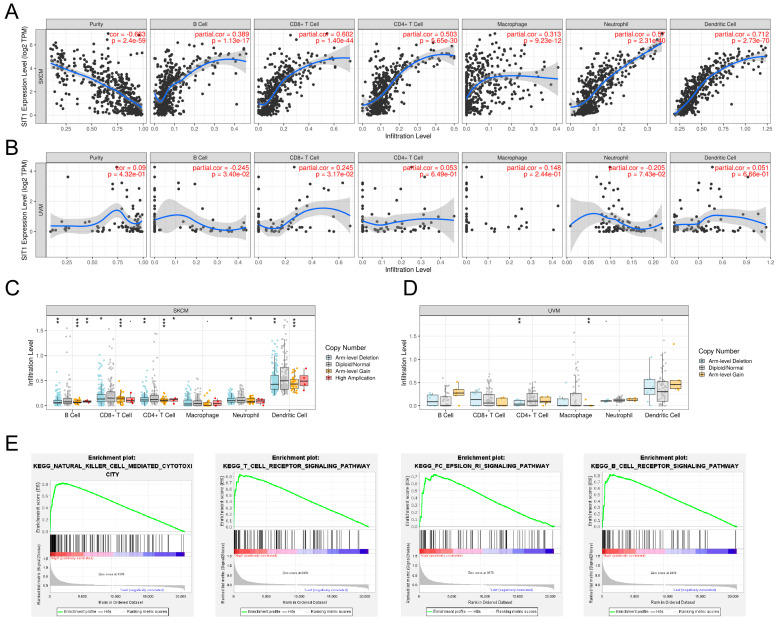
Correlation of *SIT1* expression, gene copy numbers with immune infiltration levels in melanoma and *SIT1*-associated immune signaling pathways screened by Gene Set Enrichment Analysis. (**A**) The mRNA expression of *SIT1* is uniformly positively correlated to CD8 + T cell, CD4 + T cell, B cell, dendritic cell, neutrophil, and macrophage and negatively related to tumor purity in SKCM. (**B**) *SIT1* expression has no significant correlations with infiltrating levels of CD4 + T cell, dendritic cell and macrophage infiltration, and tumor purity in UVM cell. Immune cell infiltration levels of different groups of *SIT1* copy number and in SKCM (**C**) and UVM (**D**) cohorts. *SIT1*-associated immune signaling pathways screened by GSEA in SKCM (**E**). * *p* < 0.05; ** *p* < 0.01; *** *p* < 0.001.

We studied the association between *SIT1* mRNA expression and immune cells through the TIMER website. The results showed that *SIT1* mRNA expression levels were positively correlated to various types of immune cells in many types of cancers (Figures S1 and S2). In SKCM, the *SIT1* mRNA levels were uniformly positively correlated to CD8 + T cell, CD4 + T cell, B cell, dendritic cell, neutrophil, and macrophage (Figure 2A). In contrast, there was no significant correlation between *SIT1* expression and CD4 + T cell, dendritic cell, and macrophage infiltration in UVM (Figure 2B). In addition, the immune cell infiltration levels varied between different *SIT1* gene copy numbers in head and neck squamous cell carcinoma, lung squamous cell carcinoma, stomach adenocarcinoma, and SKCM (Figure S3). In SKCM, different groups of *SIT1* gene copy numbers seemed to demonstrate different B cell, CD8 + T cell, CD4 + T cell, neutrophil, and dendritic cell infiltration levels (Figure 2C). However, no significant relevance between *SIT1* gene copy numbers and immune cell infiltration levels was found in UVM, which might be due to a relatively small sample size (Figure 2D). In addition, the infiltration levels of CD8 + T cell, B cell, dendritic cells, neutrophil, and *SIT1* mRNA expression were positively associated with SKCM patients’ overall survival (Figure S4A). In VUM, CD8 + T cell infiltration levels, neutrophil infiltration levels, and *SIT1* mRNA expression were associated with survival (Figure S4B). Since the sample size of TCGA_VUM is quite small, in order to get a reliable conclusion, we subsequently focused solely on the study of *SIT1* in SKCM patients.

The RNA-seq data of TCGA_SKCM tumor samples were separated into *SIT1*_high_ and *SIT1*_low_ groups by the medium *SIT1* mRNA level. GSEA analysis indicated that *SIT1* was positively associated with some immune-associated signaling pathways, including natural killer cell-mediated cytotoxicity (NES = 2.24, *p*_FWER_ = 0.005), FC epsilon RI signaling pathway (NES = 2.29, *p*_FWER_ = 0.003), B cell receptor signaling pathway (NES = 2.23, *p*_FWER_ = 0.005), and T cell receptor signaling pathway (NES = 2.33, *p*_FWER_ = 0.002) (Figure 2E).

**Figure 3:**
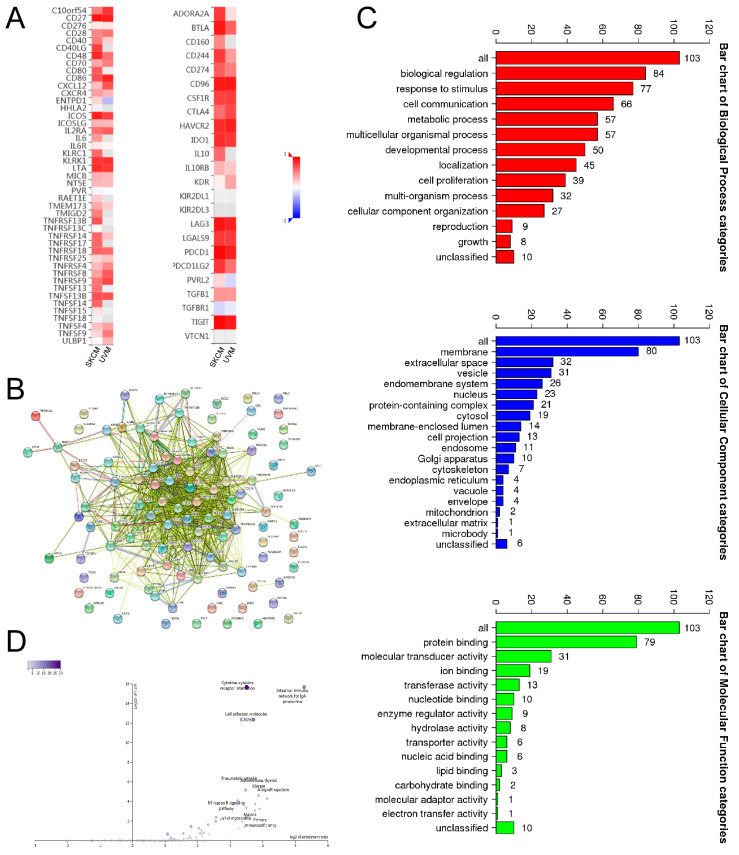
Discrimination and analysis of *SIT1*-associated immunomodulators. (**A**) The heatmaps of correlation between the *SIT1* gene and immunoinhibitors, immunostimulators in SKCM and UVM. (**B**) Protein interaction network of 58 *SIT1*-associated immunomodulators and top 50 related genes in SKCM from the STRING website. (**C**) Gene ontology analysis of the above 108 genes in SKCM. (**D**) Kyoto Encyclopedia of Genes and Genomes pathway analysis of the above 108 genes.

In order to find the key molecular factors and signaling pathways through which *SIT1* might regulate the immune response in SKCM, we screened *SIT1*-associated immunomodulators in the TISIDB database. After processing the data, we found 58 related immune genes, including 38 immunostimulators (*C10orf54*, *CD27*, *CD276*, *CD28, CD40, CD40LG, CD48, CD70, CD80, CD86, CXCL12, CXCR4, ENTPD1, ICOS, ICOSLG, IL2RA, IL6, KLRC1, KLRK1, LTA, MICB, PVR, TMEM173, TMIGD2, TNFRSF13B, TNFRSF13C, TNFRSF14, TNFRSF17, TNFRSF18, TNFRSF25, TNFRSF4, TNFRSF8, TNFRSF9, TNFSF13, TNFSF13B, TNFSF14, TNFSF15*, and *TNFSF4*) (Figure 3A) and 20 immunoinhibitors (*ADORA2A, BTLA, CD160, CD244, CD274, CD96, CSF1R, CTLA4, HAVCR2, IDO1, IL10, IL10RB, LAG3, LGALS9, PDCD1, PDCD1LG2, PVRL2, TGFB1, TGFBR1*, and *TIGIT*) (Figure 3A). The top 50 genes that were closely related to these immunomodulators were added to the immunomodulators’ protein networks as shown in Figure 3B by the STRING website. As shown in Figure 3C, most of the immunomodulators were expressed in the cell membrane to regulate the biological activity by affecting protein-binding interaction. The KEGG pathway enrichment analysis shows that the NF-κB signaling pathway is related to *SIT1*-mediated immune events (Figure 3D).

### 3.3. Association between SIT1 Expression and Immune Markers

To validate the relationship between *SIT1* and the above associated immune cells, we further studied the correlation between *SIT1* and immune markers of these immune cells in SKCM. We also studied the biomarkers of detailed T cells, such as Th1, Th2, Tfh, Th17, Treg, and exhausted T cell (Table S1). After the adjustments with the tumor purity value, the mRNA expression levels of *SIT1* were still positively correlated with the level of most biomarkers of the previously mentioned detailed immune cells in SKCM.

We observed that the biomarker expression levels of the B cell, monocyte, and Th1 cell immunomarker genes were closely correlated with *SIT1* expression in SKCM (Cor > 0.500, *p* < 0.0001) (Table S1, Figure S5). *MS4A4A* of M2 Macrophage, *CD11b*, *CCR7* of Neutrophils, *KIR2DL4*, *KIR3DL2* of natural killer cell, and *HLA-DPB1*, *HLA-DRA*, *HLA-DPA1*, *HLA-DQB1* of dendritic cell greatly correlated with *SIT1* levels in SKCM (Cor > 0.500, *p* < 0.0001).

Furthermore, we assessed the interrelationship among *SIT1* levels and CD8 + T cells, B cells, monocytes, and Th1 cell immune markers in the GEPIA dataset; the results were similar in TIMER (*p* < 0.0001; Table S1). Thus, *SIT1* may regulate tumor-specific cytotoxicity in SKCM. We also observed a close association between expression levels of *SIT1* and biomarkers of Treg and exhausted T cell, such as *FOXP3*, *CCR8*, *PDCD1*, *LAG3*, *TIM-3*, and *GZMB* (Table S1). Thus, further studies are required to determine whether *SIT1* plays a driver or passenger role during the immune escape process in SKCM microenvironments.

### 3.4. The Prognostic Implication of SIT1 and SIT1-Associated Immunomodulators in SKCM

**Figure 4:**
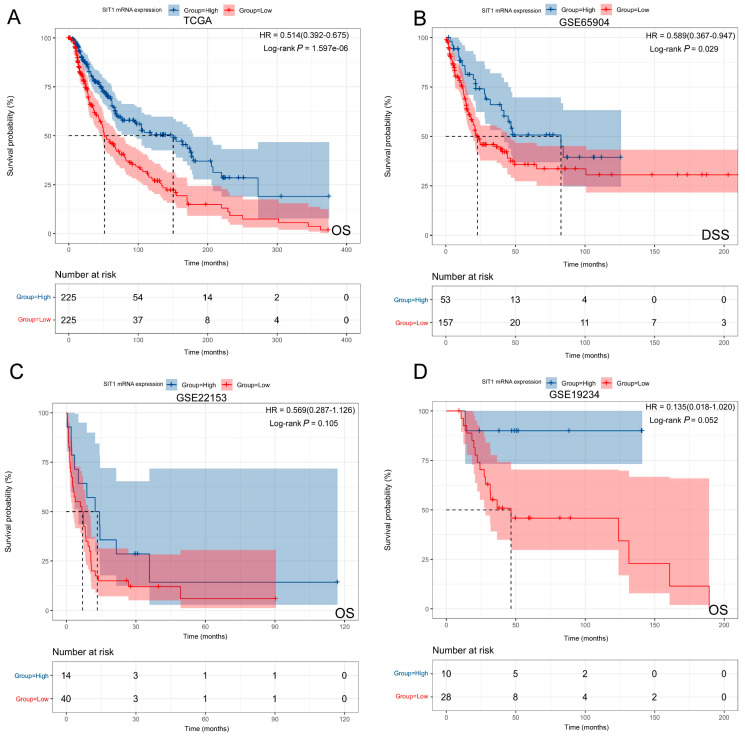
Kaplan–Meier survival analysis of *SIT1* in SKCM of different public datasets. (**A**) Kaplan–Meier survival analysis of *SIT1* in OS for the TCGA_SKCM dataset. (**B**) Kaplan–Meier survival analysis of *SIT1* in DSS for the GSE65904 dataset. Kaplan–Meier survival analysis of *SIT1* in OS for the GSE22153 (**C**) and GSE19234 (**D**) datasets. OS: overall survival; DSS: disease-specific survival.

To further evaluate the prognostic value of *SIT1* in SKCM, TCGA and several independent GEO (GSE65904, GSE22153, and GSE19234) datasets were included in the following analysis to improve the reliability of the results. Patients were divided into two groups (*SIT1*_high_ vs. *SIT1*_low_) by the median of the *SIT1* mRNA expression levels in the TCGA dataset. For GEO datasets, the superior quartile was used to replace the median owing to a small sample size. The results showed that a high *SIT1* level corresponded to a favorable prognosis in SKCM patients in TCGA (OS hazard ratio (HR) = 0.483, 95% confidence interval (CI) = 0.366–0.639, *p* < 0.001) (Figure 4A), and GSE65904 (DSS HR = 0.589, 95% CI = 0.367–0.947, *p* = 0.029) (Figure 4B) datasets. Similar tendencies were observed in GSE22153 and GSE19234 datasets, with borderline levels of significant *p* values (OS HR = 0.569 and 0.135, 95% CI = 0.287–1.126 and 0.018–1.020, *p* = 0.105 and = 0.052, respectively) (Figure 4C,D). The effects were the same for DFS in the GSE65904 dataset (Figure S6). These results indicate that *SIT1* expression is strongly associated with the prognosis of SKCM patients.

**Figure 5:**
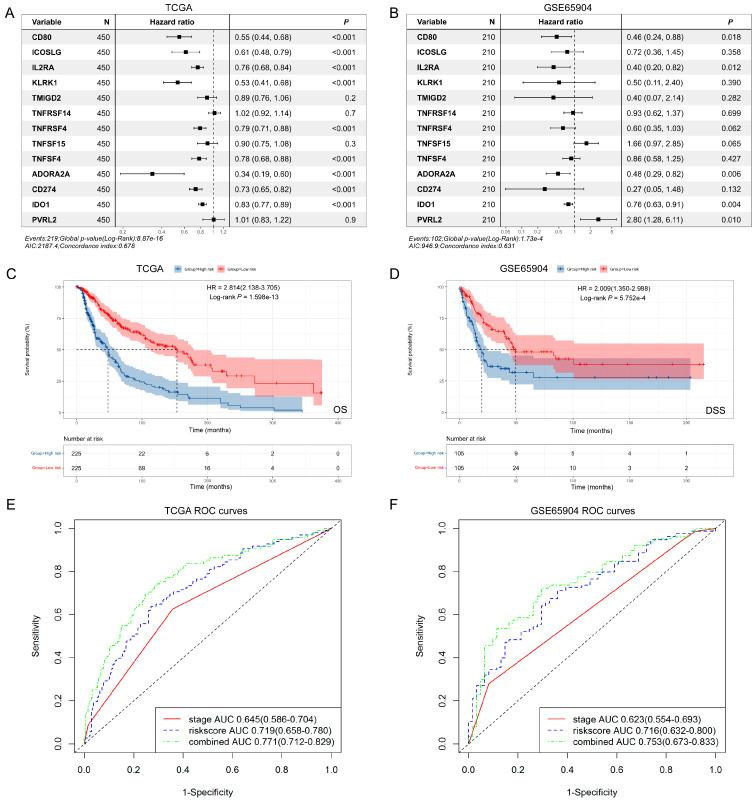
The construction of prognostic signatures for SKCM based on 58 *SIT1*-associated immunomodulators and *SIT1*. The hazard ratios of the prognostic signature genes are shown in the forest plots for SKCM patients in the TCGA (**A**) and GSE65904 (**B**) datasets. Kaplan–Meier curves of different risk groups in the TCGA (**C**) and GSE65904 (**D**) datasets for SKCM. Time-dependent receiver operating characteristic curves at 3 years for SKCM in the TCGA (**E**) and GSE65904 (**F**) datasets.

**Figure 6:**
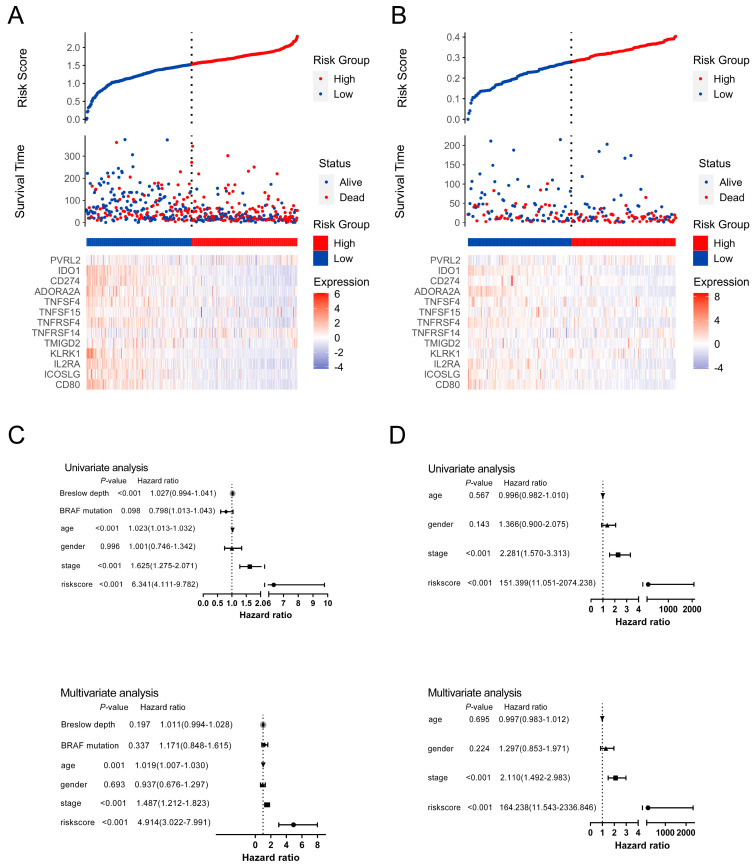
Prognostic values of the signature risk score in the TCGA-SKCM and GSE65904 cohorts. Distribution of signature gene expression profiles along with survival statuses in different risk score groups in the TCGA (**A**) and GSE65904 (**B**) datasets. Univariate and multivariate Cox regression analyses of the signature risk score in the TCGA (**C**) and GSE65904 (**D**) datasets.

Then, we used the TCGA dataset with 450 patients as the discovery cohort to build a *SIT1*-associated immunomodulator prognostic signature in SKCM patients through the LASSO Cox regression analysis. An optimal 13-gene prognostic signature was made after the previous process. The biological functions of signature genes are shown in Table S2. The signature risk scores were equal to the sum of the products of expression value and coefficient of each gene. We choose the GSE65904 dataset with a relatively large sample size (210) as the validation cohort to measure the prognostic value of the signature-based risk score. The associations between the risk score and clinicopathological features were analyzed first. In the TCGA dataset, there were more patients older than 60 (*p* = 0.047) and with a Breslow depth larger than 2 cm (*p* < 0.0001) in high-risk patients. More patients were at a low Clark level (I–III, *p* = 0.002) and T stage (T1–T2, *p* < 0.0001) in the low-risk population. High-risk patients seemed to have more death than low-risk patients (*p* = 0.0002) (Table S3). However, there were no significant differences in age, gender, stage, and death between different risk groups in the GSE65904 dataset (Table S4). As shown in Figure 5A,B, most signature genes were independently associated with OS. Then, we used the log-rank test to study the association of the risk score with survival in the TCGA dataset; as we expected, the high-risk patients had significantly shorter survival than low-risk patients (log-rank test, *p* < 0.001) (Figure 5C). The same tendency was confirmed in the GSE65904 dataset (log-rank test, *p* < 0.001) (Figure 5D). The area under the curve (AUC) values of time-dependent receiver operating characteristic curves (ROC) at 3 years for the risk score and stage in the TCGA dataset were 0.719 (95% CI = 0.658–0.780) and 0.645 (95% CI = 0.586–0.704), respectively. An AUC of 0.771 (95% CI = 0.712–0.829) was achieved when the risk score and stage were combined (Figure 5E). The AUC values of the risk score, stage, and combined factors in the GSE65904 dataset were 0.716 (95% CI = 0.632–0.800), 0.623 (95% CI = 0.554–0.693), and 0.753 (95% CI = 0.673–0.833), respectively (Figure 5F). Figure 6A,B shows the distribution of signature gene expression profiles and survival statuses in different risk score groups for SKCM in both datasets. Figure 6C,D shows that a high-risk score was significantly associated with increased mortality risk in SKCM patients in the univariate Cox regression models in both datasets [HR = 6.341 and 151.399, 95% CI = 4.111–9.782 and 11.051–2074.238, *p* < 0.001 and < 0.001, respectively]. Similar results were found in multivariate Cox regression after adjusting for age, gender, stage, etc. (HR = 4.914 and 164.238, 95% CI = 3.022–7.991 and 11.543–2336.846, *p* < 0.001 and < 0.001, respectively).

### 3.5. The Predictive Value of SIT1-Associated Immunomodulators Signature for the Efficacy of Immunotherapy on Melanoma Patients

**Figure 7:**
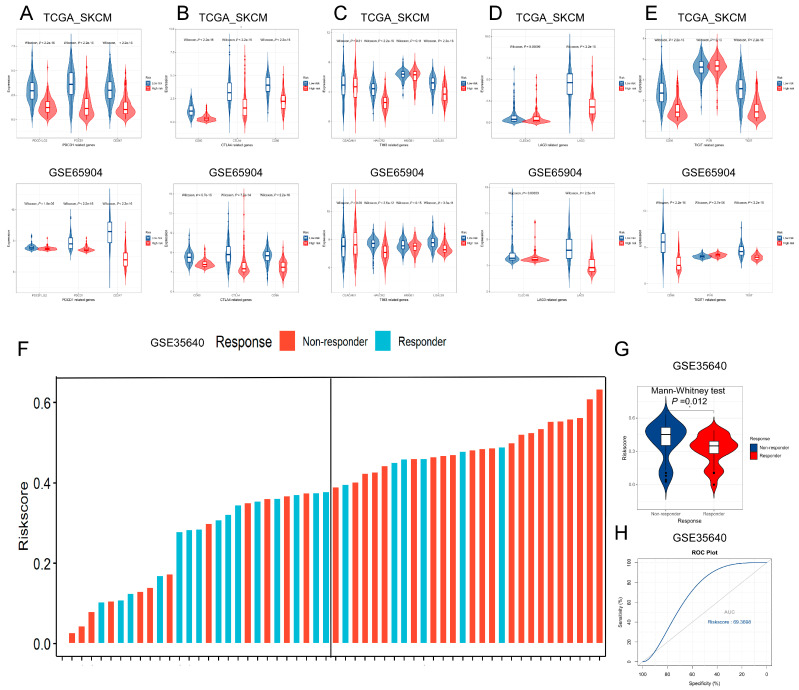
The association between signature risk score and efficacy of immunotherapy for SKCM patients. (**A**–**E**) Comparison of hot immune pathway key genes (PD-1/PD-L1 signaling pathway-related genes, CTLA4/CD80-86 signaling pathway-related genes, TIM3/TIM3L signaling pathway-related genes, LAG3/LAG3L signaling pathway-related genes, TIGIT/CD96 signaling pathway-related genes.) expression between low- and high-risk groups based on the TCGA (**A**) and GSE65904 datasets (**B**). (**C**) Responder melanoma patients from the GSE35640 dataset show lower risk scores than other patients. (**D**) Melanoma patients with high-risk scores from the GSE35640 dataset contain seldom responders. (**E**) ROC curve showing predictable efficacy of immunotherapy based on our signature risk score for SKCM patients in the GSE35640 dataset (AUC = 0.694). (**F**) Responder melanoma patients from the GSE35640 dataset show lower risk scores than other patients. (**G**) Melanoma patients with high-risk scores from the GSE35640 dataset contain seldom responders. (**H**) ROC curve showing predictable efficacy of immunotherapy based on our signature risk score for SKCM patients in the GSE35640 dataset (AUC = 0.694).

As the risk scores were calculated from the expression of *SIT1*-associated immunomodulators, which may indicate the immune activity, we further tested if it could predict the efficacy of the immunotherapy response. Current hot immunotherapy-targeted gene expressions in melanoma patients were compared between different risk groups from the datasets TCGA_SKCM and GSE65904. The low-risk population was found to have higher expression of PD-1/PD-L1 signaling pathway- (*PDCD1LG2*, *CD274*, and *PDCD1*) (Figure 7A), CTLA4/CD80-86 signaling pathway- (*CD80*, *CTLA4*, and *CD86*) (Figure 7B), TIM3/TIM3L signaling pathway- (*HAVCR2* and *LGALS9*) (Figure 7C), LAG3/LAG3L signaling pathway- (*LAG3* and *CLEC4G*) (Figure 7D), and TIGIT/CD96 signaling pathway- (*TIGIT* and *CD96*) (Figure 7E) -related genes than the high-risk population. This indicates that immunotherapy targeted for the above signaling pathways may have a better response in low-risk patients.

Melanoma patients from the GSE35640 dataset were used to test the above hypothesis further. Those patients had exhibited different treatment response s(response and non-response) after receiving MAGE-A3 cancer immunotherapy. We divided the patients into two groups by the treatment responses and compared the risk scores between those groups. The results showed that the responder group had a significantly lower risk score than the other group (Figure 7F). From Figure 7G, we observed there are seldom responders in high-risk patients. The signature risk scores show a good predictability for the patients’ response to immunotherapy in the GSE35640 dataset with an AUC reaching 0.694 (Figure 7H).

**Figure 8:**
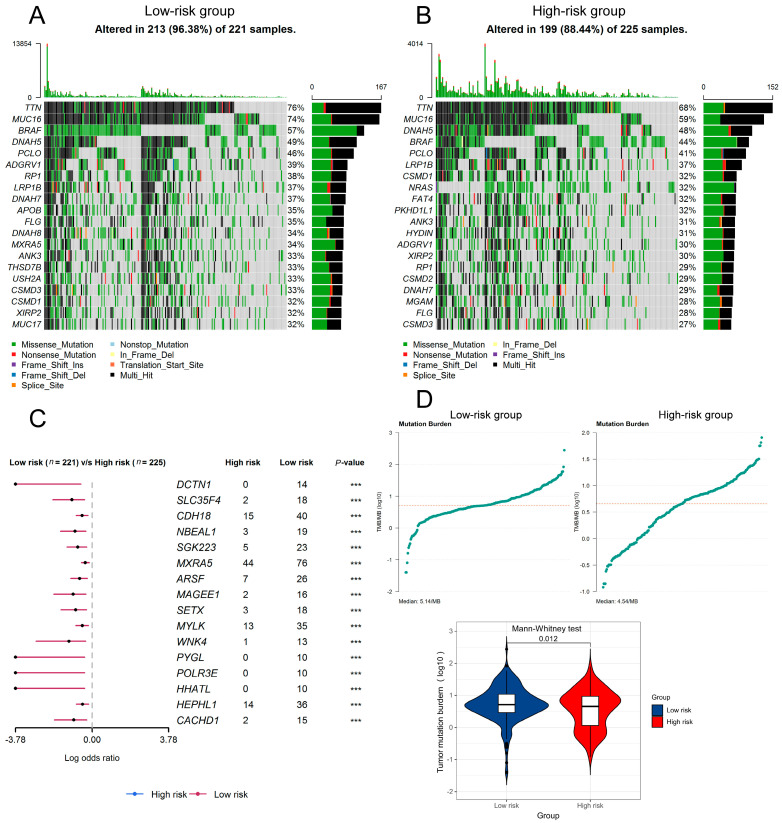
Analysis of tumor mutation in low- and high-risk melanoma patients. (**A**) Mutation landscape of low-risk melanoma patients. (**B**) Mutation landscape of high-risk melanoma patients. (**C**) Comparison of prevalent mutation frequency between low-risk group and high-risk group. (**D**) Different tumor mutation burden in low- and high-risk groups.

Since high TMB also indicates a better immunotherapy response, we wondered if there is any difference between high- and low-risk patients. As seen in Figure 8A,B, the low-risk population seems to have a higher mutation frequency of all genes (96.38% vs. 88.44%). There were also more prevalent mutations in low-risk than high-risk populations (Figure 8C). As expected, the low-risk group also had a significantly higher TMB (5.14 vs. 4.54/MB, *p* = 0.012) (Figure 8D) than the other group.

These results indicate a good predictive value of *SIT1*-associated immunomodulators signature for the efficacy of immunotherapy on melanoma patients.

### 3.6. Construction of Nomogram

**Figure 9:**
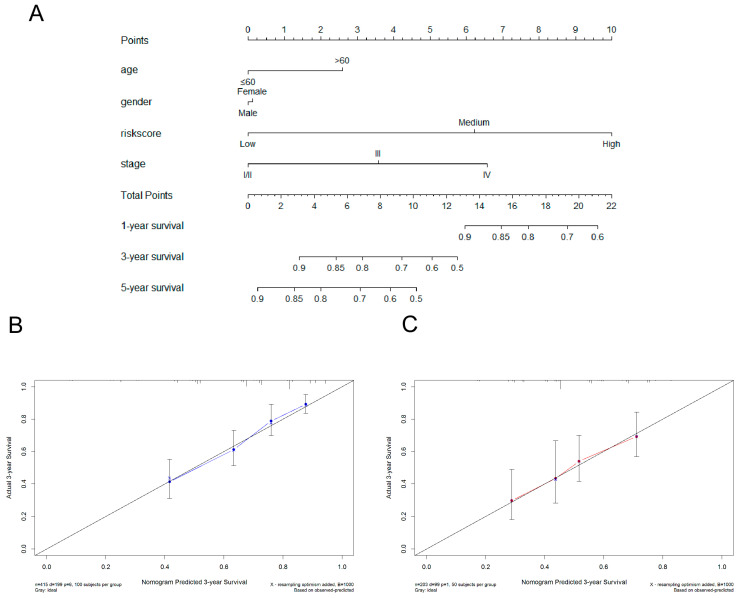
Establishment and validation of the prognostic nomogram combining signature risk scores and important risk factors in SKCM. (**A**) A nomogram for predicting 1-, 3-, and 5-year survival probabilities for SKCM patients. The calibration curves of 3-year survival in the TCGA (**B**) and GSE65904 (**C**) datasets. The 45° dashed line represents a close match between predicted and real probabilities. However, we did not include other important risk factors (etc. T stage, N stage, M stage, Breslow depth, and BRAF/NRAS mutations) in our predictive model because they were unavailable in the TCGA or GSE65904 datasets, which might significantly decrease the accuracy of the nomogram.

A nomogram was built by combing signature risk scores and other important characteristics (age, gender, and stage) from the TCGA dataset to predict a patient’s prognosis (Figure 9A), whereas the GSE65904 dataset was used to test its accuracy. Our prognostic nomogram reached a C-index of 0.708 (95% CI = 0.632–0.784) in the TCGA dataset and 0.647 (95% CI = 0.537–0.757) in the GSE65904 dataset, suggesting an acceptable and stable predictability. As shown in Figure 9B,C, the predicted 3-year survival probability closely matched the real 3-year survival probability in the TCGA and GSE65904 datasets. Similar results were found in calibration curves for 1- and 5-year survival (Figure S7A–D).

## 4. Discussion

Immunotherapy by targeting checkpoints has dramatically improved the prognosis of SKCM patients. However, only a small percentage of melanoma patients could benefit from the treatment. Previous studies have found that immune cell infiltration plays an important role in immunotherapy [33,34,35,36] and the function of different immune cells varies in anti-tumor immune response [37]. In addition, the eradication of tumors by the immunity response is a complicated multi-step process [38]; thus, failure in any one of those critical steps may decrease the treatment efficacy. However, the mechanism underlying immunotherapy failure is still undefined. Therefore, it is still important to improve the efficiency of immunotherapeutic drugs and the identification of patients who could benefit from the treatment. Individualized treatment strategies in SKCM patients require biomarkers or models with good predictable accuracy on the prognosis or immunotherapy efficacy.

The signaling threshold regulating transmembrane adaptor 1 (SIT1), encoded by the *SIT1* gene, was not identified until 1999. Previous studies have shown that SIT1 regulated human T cell activation by recruiting the SH2 domain-containing tyrosine phosphatase SHP2 via an immunoreceptor tyrosine-based inhibition motif [39]. Moreover, SIT1 is able to inhibit the TCR-mediated activation of protein kinase C [20]. However, the mechanism by which *SIT1* regulates human immunity is not fully understood. In addition, there are few studies showing the relationship between *SIT1* and cancer biology or immunity. The immunity implications of the *SIT1* gene in malignant tumors remain mostly unknown.

In this study, we found that *SIT1* mRNA expression levels varied between different tumors and adjacent normal tissues. Our results also demonstrate that *SIT1* mRNA expression levels were positively correlated to various types of immune cells in many types of cancers, including SKCM. *SIT1* expression levels also strongly correlate with the expression of the CD8 + T cell, Th1 cell, Treg, exhausted T cell, monocyte, and B cell biomarkers in SKCM. GSEA analysis indicates that *SIT1* is positively correlated with T cell, B cell, and natural killer cell-related pathways. KEGG pathway analysis reveals that the NF-κB signaling pathway might be involved in the *SIT1*-mediated immune response. These results strongly indicate that *SIT1* may influence SKCM immunity by affecting T cell, B cell, and natural killer cell activity. *SIT1* expression is also significantly associated with SKCM prognosis. We built a novel prognostic immune gene signature by *SIT1*-associated immunomodulators, which shows a better predictive value than previously reported gene signatures (Figure S9). A nomogram was constructed by combing signature risk scores and other important characteristics (age, gender, and stage) from the TCGA dataset to predict a patient’s prognosis (Figure 9A), whereas the GSE65904 dataset was used to test its accuracy. Our prognostic nomogram showed acceptable and stable predictability.

Previous studies reported that SIT1 is mainly expressed in the plasma membrane of T- and B-lymphocytes [19,20,40,41,42]. However, a lower SIT1 protein expression was also found in the plasma of a very small percentage of melanoma cells using the immunohistochemistry method through the Protein Atlas website (Figure S8). There was also a study that reported that the SIT1 protein was detected in extracellular exosomes, but its biological function is still unknown. It is possible that a few melanoma cells express SIT1 protein and release it into the tumor microenvironment by exosome to regulate tumor immune response. Since *SIT1* mRNA expression levels are negatively correlated to tumor purity in many types of cancers in our analysis and its protein is mainly expressed in T- and B-lymphocytes, a relatively higher *SIT1* mRNA expression in tumor microenvironments represent a high degree of lymphocytes infiltration. It seems logical for patients with high *SIT1* mRNA expression to have a good prognosis. However, many studies have reported that high degrees of lymphocyte infiltration does not mean an activated immune response [43]. Thus, the biological function of *SIT1* in melanoma immunity is complicated; it would be very important and interesting to focus on the subject in the future.

In order to formulate suitable personalized treatment strategies, it is important to improve the predictive accuracy of immunotherapy responses in melanoma patients. The success of immune treatment by checkpoint blockade and adoptive T cell therapy demonstrates the vital role of CD8 + T cells in mediating anti-tumor responses [14]. Most patients cannot continuously benefit from immunotherapy because of dysfunction or deficiency of CD8 + T cells. It is essential to preserve the anti-tumor activity of CD8 + T cells for the prolonged survival of SKCM patients. Thus, it is logical to deduce that the combinational use of different immune-inhibitory pathway inhibitors may be more effective in melanoma patients. Several studies have demonstrated the encouraging results of combinational immunotherapy. Nivolumab-plus-ipilimumab immunotherapy significantly prolonged the OS of melanoma patients compared with nivolumab or ipilimumab monotherapy [16]. Other combinations of inhibitors, such as anti-PD-1 plus LAG3 blockade, have reached a 16% objective response rate and 45% disease control rate in melanoma patients following progression after prior PD-1 blockade [17]. A previous study has also indicated that anti-PD-L1 and anti-TIGIT antibodies synergistically enhanced tumor-infiltrating lymphocyte activity in melanoma [18]. Low-risk melanoma patients, predicted by our signature, exhibited an elevated expression of inhibitory receptors and ligands as well as high TMB, which should be more sensitive to mono or combined immunotherapy. As we expected, the signature risk score has a good predictive accuracy of MAGE-A3 cancer immunotherapy response in melanoma patients.

Open access to high-dimensional datasets and advanced bioinformatics algorithms helps us to screen for more reliable and robust SKCM biomarkers more easily than in the past. Many prognostic gene expression-based signatures have been built by different teams researching SKCM recently [22,44,45,46,47,48]. Among the prognostic signatures, the majority was built based on immunity-related genes [49,50]. The genes of prognostic signature from Hu et al. were related to IFNγ. Liu et al. developed a prognostic immune-related gene signature for 10 distinct genes in the TCGA_SKCM database. With the signature, melanoma patients in the low-risk group showed high TMB and a good response to MAGE-A3 immunotherapy. Similar studies constructed a prognostic signature with M2 macrophage or CD8 + T cell infiltration-related coexpressed genes, which also showed good prognostic ability. Metastasis-related genes were also used to build a prognostic marker by Wan et al. In order to test our gene signature, we compare it with the above existing gene signatures and confirmed it showed better predictive value than others (Figure S9).

Our prognostic signature consisted of thirteen genes, which were *CD80, ICOSLG, IL2RA, KLRK1, TMIGD2, TNFRSF14, TNFRSF4, TNFSF15, TNFSF4, ADORA2A, CD274, IDO1*, and *NECTIN2*. Among them, *CD80, ICOSLG, IL2RA, KLRK1, TNFRSF4, TNFSF4, ADORA2A, CD274*, and *IDO1* were determined to be favorable biomarkers for OS prognosis, whereas *TMIGD2, TNFRSF14, TNFSF15*, and *NECTIN2* were determined to be unfavorable biomarkers. The most favorable biomarkers have been reported to mainly regulate the T cell biological process. *CD274* encodes PD-L1, which is well known for its suppressive role in blocking T-cell activation and has therefore already been used as a target in cancer immunotherapy. Many studies have proven its huge value in treating cancer patients by targeting PD-L1 [51]. CD80 is a membrane receptor that is activated by the binding of CD28 or CTLA-4 and induces T-cell proliferation and cytokine production [52,53]. ICOSLG acts as a costimulatory signal for T-cell proliferation and cytokine secretion [54]. It also induces B-cell proliferation and differentiation into plasma cells [55]. IL2RA is involved in the regulation of immune tolerance by controlling regulatory T cell activity and inducing cell death of T cells [56]. KLRK1 can result in the activation of NK and T cells, which have been chosen as a therapeutic target for the treatment of immune diseases and cancers [57,58]. TNFRSF4 binds with TNFSF4 to co-stimulate T-cell proliferation then functions in T-cell antigen-presenting cell interactions and mediates the adhesion of activated T cells to endothelial cells [59]. IDO1 regulates T lymphocyte division, apoptosis, and differentiation [60]. ADORA2A is a guanine nucleotide-binding protein-coupled receptor that can increase intracellular cAMP levels. It plays an important role in many biological functions including immune function [61,62]. The most unfavorable biomarkers have also been reported to be linked with T-cell activity. TMIGD2 enhances T-cell proliferation and cytokine production via an AKT-dependent signaling cascade [63]. TNFRSF14 functions in signal transduction pathways that activate T-cell immune response [59]. TNFSF15 is not expressed in either B or T cells, so it is probably expressed in melanoma cells. It can activate NF-κB and MAP kinases and facilitates the differentiation and polarization of macrophages [64]. NECTIN2 can be either a costimulator or a coinhibitor of T-cell function, depending on the competitive binding receptors [65]. Upon binding to CD226, it stimulates T-cell proliferation and cytokine. Upon interaction with PVRIG, it inhibits T-cell proliferation. The gene signature was only constructed using immunomodulators, so it may be suitable for predicting cancer immunotherapy response, however, it is not perfect for providing a prognostic signature for melanoma patients owing to that immunity is only one of the key factors regulating cancer prognosis.

There are several limitations in our present study. Since our results were calculated from public datasets, publication bias in these datasets may increase the inaccuracy of our results. Besides, the present study was a retrospective analysis, which needs to be validated in the future by large and prospective studies. At last, the complicated molecular mechanisms of *SIT1*-medicated tumor immunity have not been addressed in this study.

## 5. Conclusions

Our research provides evidence that *SIT1* may regulate SKCM tumor immune microenvironments. The *SIT1*-associated immunomodulator signature risk scores were independent risk factors of OS and the efficacy of immunotherapy in SKCM. In addition, the nomogram combining the risk score with other important variables (stage, gender, and age), shows acceptable C-index value and well-matched calibration curves in discovery and validation cohorts. It is foreseeable that the predictive accuracy will be improved along with the development of high-dimensional databases and bioinformatic methods, thus beneficial individualized treatment strategies will be accessible in the near future.

## Data Availability

All datasets analyzed in the present study are open access. These data can be found in the websites below: Cancer Genome Atlas (https://portal.gdc.cancer.gov/) (accessed on 15 April 2022), GDC hub of UCSC Xena website (http://xena.ucsc.edu/public) (accessed on 15 April 2022) and GEO database (https://www.ncbi.nlm.nih.gov/geo/query/acc.cgi?acc=GSE65904 (accessed on 15 April 2022); https://www.ncbi.nlm.nih.gov/geo/query/acc.cgi?acc=GSE22153 (accessed on 15 April 2022); https://www.ncbi.nlm.nih.gov/geo/query/acc.cgi?acc=GSE19234 (accessed on 15 April 2022); https://www.ncbi.nlm.nih.gov/geo/query/acc.cgi?acc=GSE98394 (accessed on 15 April 2022); https://www.ncbi.nlm.nih.gov/geo/query/acc.cgi?acc=GSE35640) (accessed on 15 April 2022).

## Supplementary Materials

The following supporting information can be downloaded from: https://www.mdpi.com/article/10.3390/jpm13010013/s1, Figure S1. Correlation between *SIT1* expression levels and immune cell subsets. The correlation heatmap indicates immune cell types significantly associated with *SIT1* expression levels in most cancer cohorts; Figure S2. Associations between *SIT1* mRNA and immune cell infiltration levels. Association between *SIT1* mRNA and immune cell infiltration levels in cancer cohorts except SKCM and UVM; Figure S3. Associations between *SIT1* gene copy numbers and immune cell infiltration levels. Association between *SIT1* copy numbers and immune cell infiltration levels in cancer cohorts except SKCM and UVM. * *p* < 0.05; ** *p* < 0.01; *** *p* < 0.001.; Figure S4. Kaplan–Meier curves describe the association between survival and six tumor immune cells as well as the *SIT1* gene via the TIMER web-based tool (https://cistrome.shinyapps.io/timer/) (accessed on 15 April 2022). Kaplan–Meier curves describe the association between survival and six tumor immune cells as well as the *SIT1* gene for SKCM (A) and UVM (B); Figure S5. The correlation between the expression level of *SIT1* and immune marker genes in SKCM. Markers include CD8A and CD8B of CD8 + T cell; CD19 and CD79A of B cell; CD86 and CSF1R of monocytes; TBX21, STAT4, STAT1, IFNG, and TNF of Th1 cell. (A–D) Scatterplots of correlations between *SIT1* expression and gene markers of CD8 + T cell (A), B cell (B), monocytes (C), and Th1 cell (D) in SKCM; Figure S6. Kaplan–Meier survival analysis of *SIT1* in SKCM of GSE65904 dataset. Kaplan–Meier survival analysis of *SIT1* for DFS in the GSE65904 dataset. DFS: disease-free survival; Figure S7. Establishment and validation of the prognostic nomogram in SKCM with the inclusion of the risk score. The calibration curve of 1-year (A) and 5-year survival (B) in the TCGA datasets. The calibration curve of 1-year (C) and 5-year survival (D) in the GSE65904 dataset. The 45º dashed line represents a perfect uniformity between nomogram-predicted and real possibilities; Figure S8. Different *SIT1* protein expressions in SKCM tumor cells. Positive (A) and negative (B) expression of *SIT1* protein in SKCM tumor cells; Figure S9. Comparisons of the predictivity between this study and other studies. (A) Predictivity of our risk score in melanoma patients’ 1-year, 3-year, and 5-year survival from the TCGA_SKCM and GSE65904 datasets. (B) Predictivity of Yuan’s study in melanoma patients’ 1-year, 3-year, and 5-year survival from the TCGA_SKCM and GSE65904 datasets. (C) Predictivity of Liu’s study in melanoma patients’ 1-year, 3-year, and 5-year survival from the TCGA_SKCM and GSE65904 datasets. (D) Predictivity of Hu’s study in melanoma patients’ 1-year, 3-year, and 5-year survival from the TCGA_SKCM and GSE65904 datasets. (E) Predictivity of Tian’s study in melanoma patients’ 1-year, 3-year, and 5-year survival from the TCGA_SKCM and GSE65904 datasets. (F) Predictivity of Song’s study in melanoma patients’ 1-year, 3-year, and 5-year survival from the TCGA_SKCM and GSE65904 datasets. (G) Predictivity of Wan’s study in melanoma patients’ 1-year, 3-year, and 5-year survival from the TCGA_SKCM and GSE65904 datasets. (H) Predictivity of Yan’s study in melanoma patients’ 1-year, 3-year, and 5-year survival from the TCGA_SKCM and GSE65904 datasets; Figure S10. Relation between risk score and immune cell infiltration. (A–F) Correlation between risk score and the infiltrating number of CD8 T cells in melanoma patients from the SKCM_TCGA dataset by analysis with TIMER (A), QUANTISEQ (B), MCPCOUNTER (C), XCELL (D), EPIC (E), and CIBESORT (F). ESTIMATE analysis of immune score (G) and microenvironment score (H) shows a significant difference between high-risk and low-risk melanoma patients. (I) Stromal score shows a borderline significant difference between high-risk and low-risk melanoma patients; Table S1: correlation analysis between *SIT1* and markers of immune cells in SKCM of TIMER and GEPIA; Table S2: functions of the genes included in the prognostic signatures; Table S3: comparison of clinicopathological features between the low- and high-risk subgroups of the TCGA_SKCM dataset; Table S4: comparison of clinicopathological features between the low- and high-risk subgroups of the GSE69540 dataset.
