# Supplementary material for: Discovery and Validation of a SIT1-Related Prognostic Signature Associated with Immune Infiltration in Cutaneous Melanoma
Source: J Pers Med. 2022 Dec 21;13(1):13. doi: 10.3390/jpm13010013 (PMC9866779; doi:10.3390/jpm13010013)
Supplement: Supplementary file 1 [file jpm-13-00013-s001.zip › jpm-2044616-supplementary.pdf]

Supplementary Figure legend

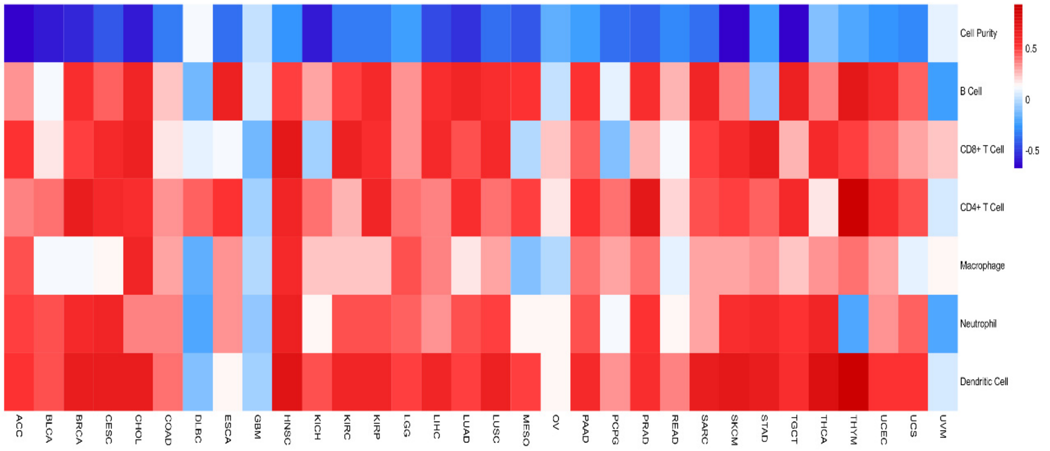

Figure S1. Correlation between *SIT1* expression levels and immune cell subsets. The correlation heatmap indicated immune cell types significantly associated with *SIT1* expression levels in most cancer cohorts.

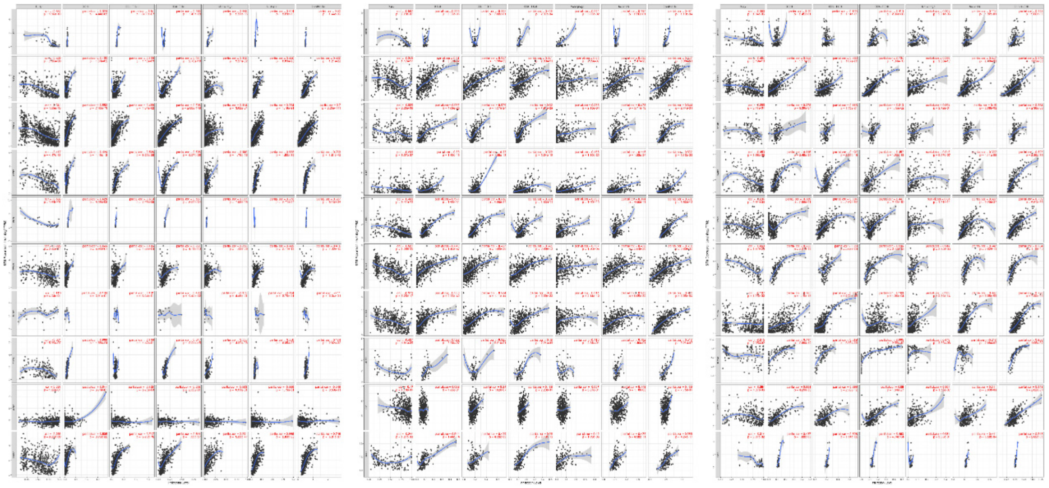

Figure S2. Associations between *SIT1* mRNA and immune cell infiltration levels. Association between *SIT1* mRNA and immune cell infiltration levels in cancer cohorts except SKCM and UVM.

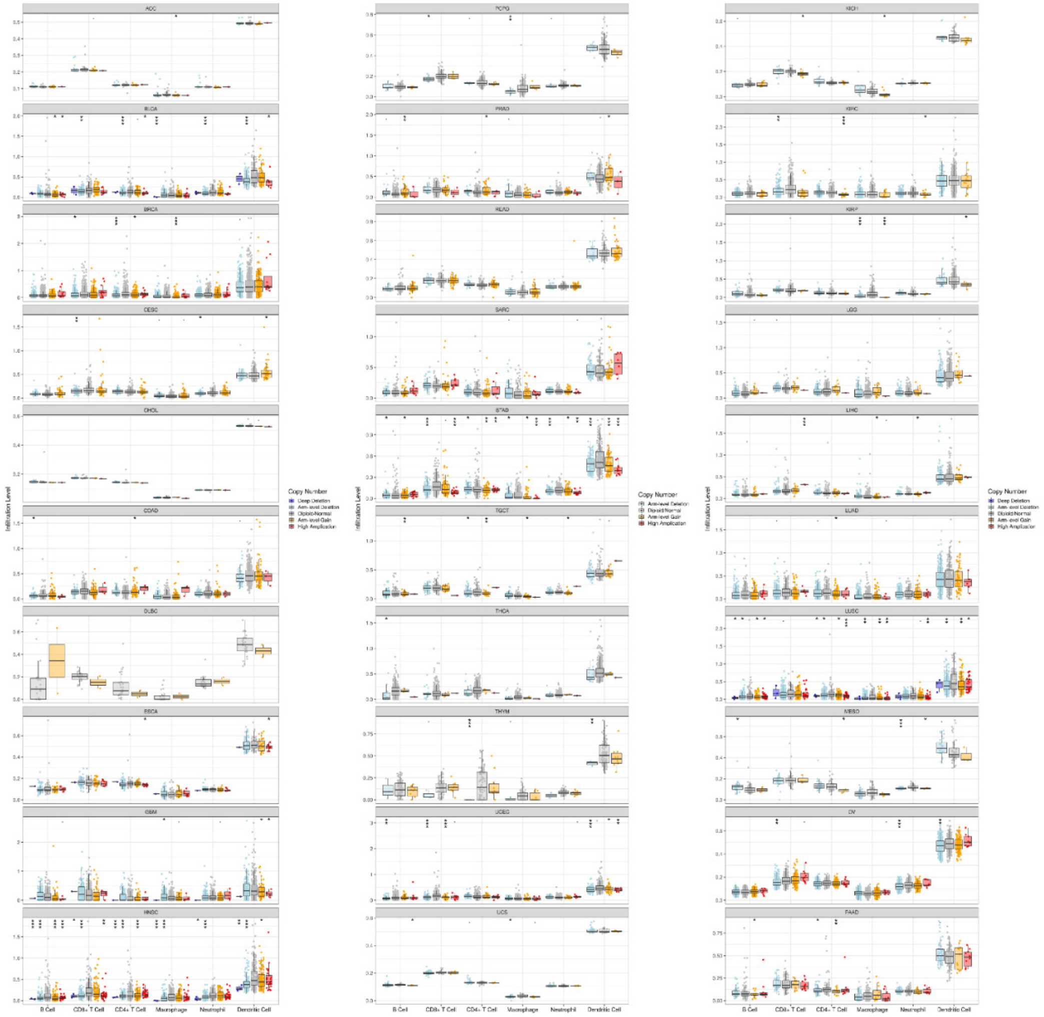

Figure S3. Associations between *SIT1* gene copy numbers and immune cell infiltration levels. Association between *SIT1* copy numbers and immune cell infiltration levels in cancer cohorts except SKCM and UVM. * $P < 0.05$; ** $P < 0.01$; ***$P < 0.001$.

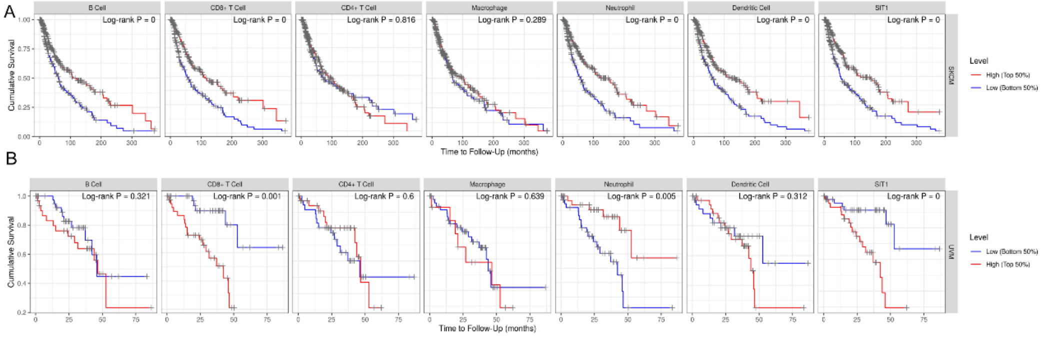

Figure S4. Kaplan-Meier curves described the association between survival and six tumor immune cells as well as the *SIT1* gene via the TIMER web-based tool (https://cistrome.shinyapps.io/timer/). Kaplan-Meier curves described the association between survival and six tumor immune cells as well as the *SIT1* gene for SKCM (A) and UVM (B).

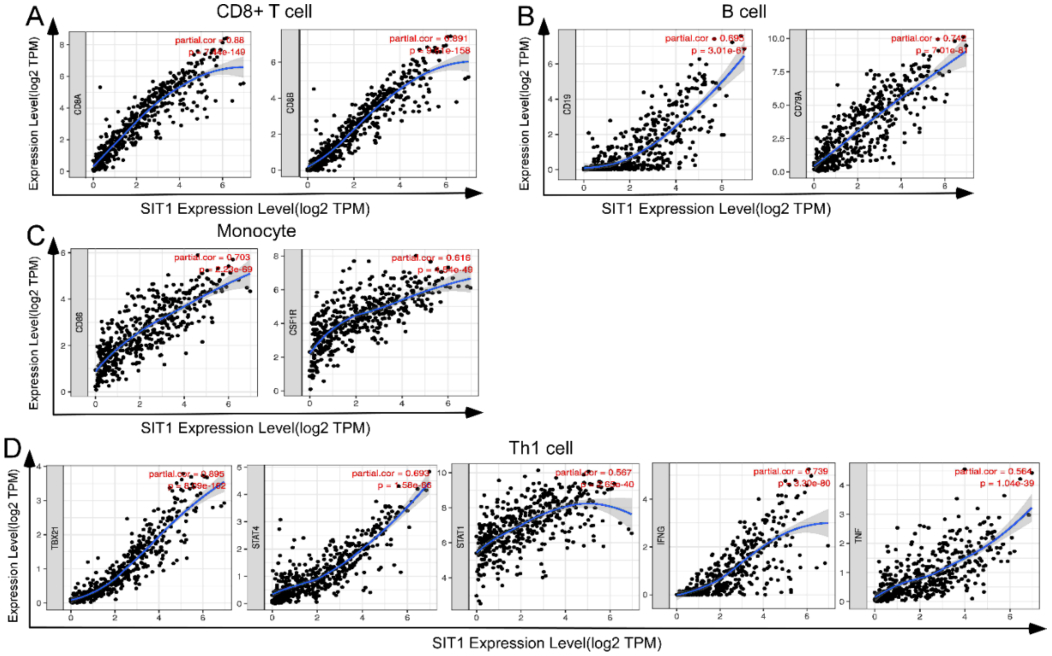

Figure S5. The correlation between the expression level of *SIT1* and immune marker genes in SKCM. Markers include CD8A and CD8B of CD8+ T cell; CD19 and CD79A of B cell; CD86 and CSF1R of monocytes; TBX21, STAT4, STAT1, IFNG and TNF of Th1 cell. (A–D) Scatterplots of correlations between *SIT1* expression and gene markers of CD8+ T cell (A), B cell (B), monocytes (C) and Th1 cell (D) in SKCM.

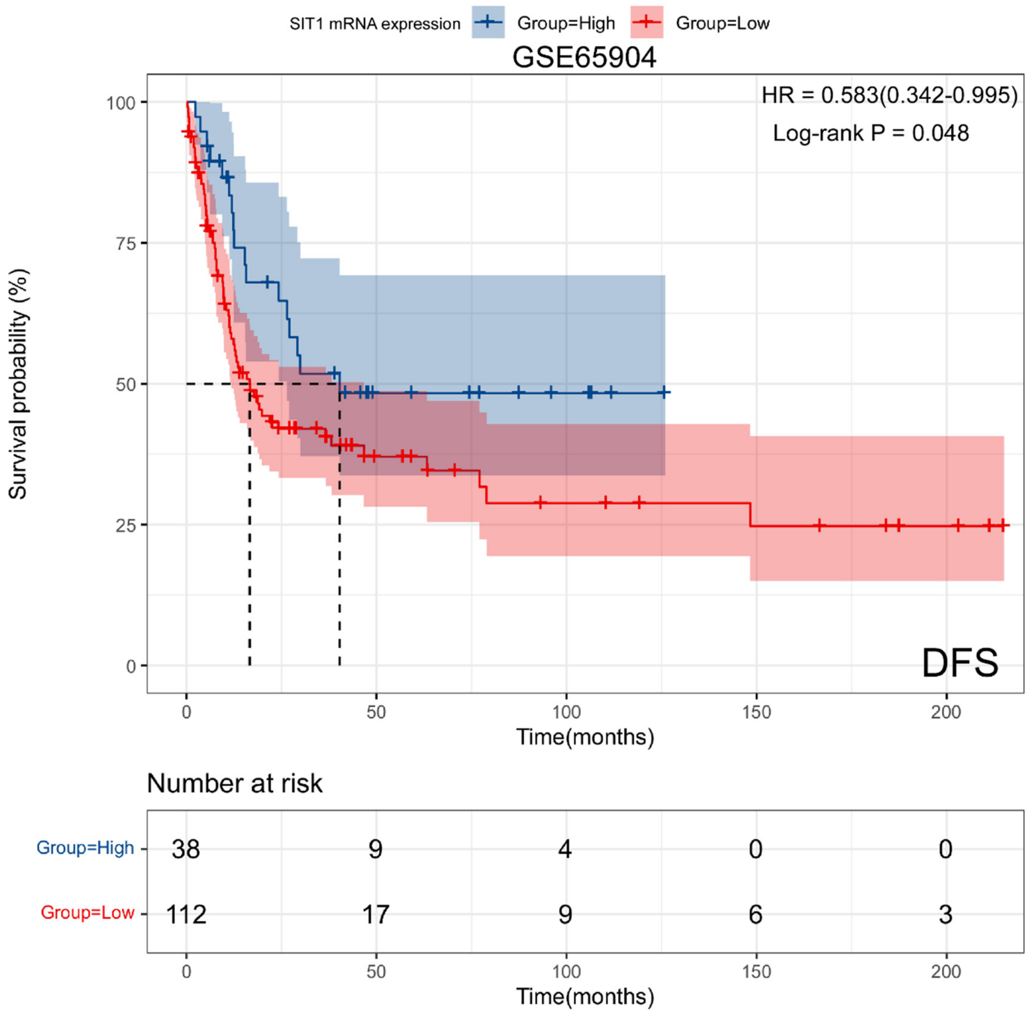

Figure S6. Kaplan-Meier survival analysis of *SIT1* in SKCM of GSE65904 dataset. Kaplan-Meier survival analysis of *SIT1* in DFS of GSE65904 dataset. DFS: disease-free survival.

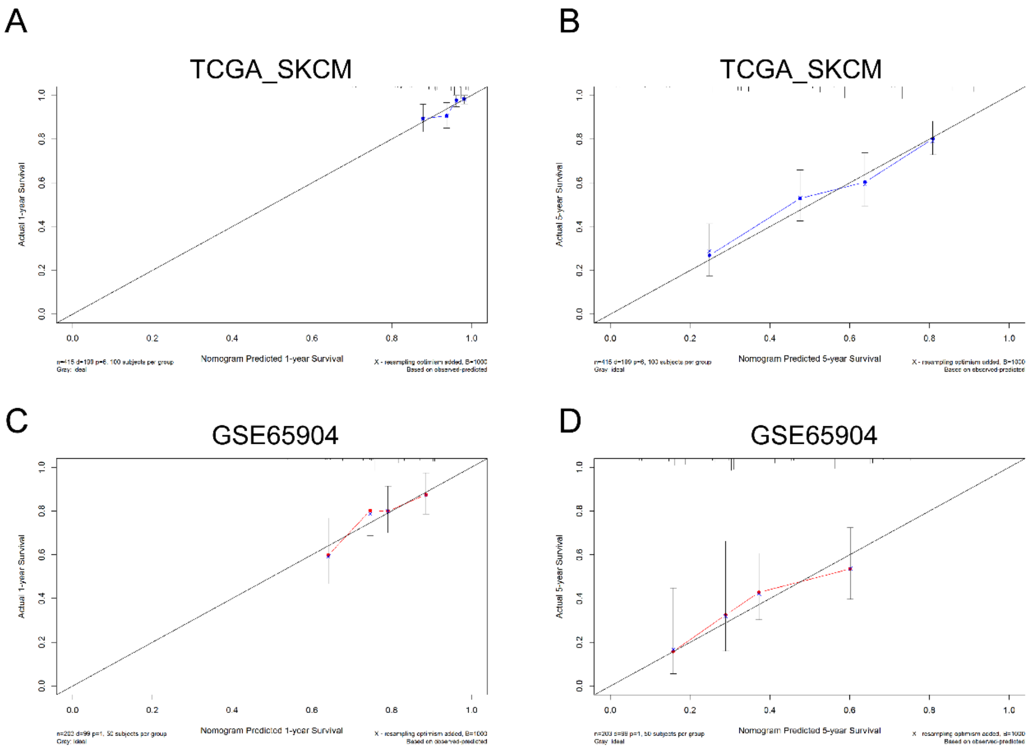

Figure S7. Establishment and validation of the prognostic nomogram in SKCM with the inclusion of the risk score. The calibration curve of 1-year (A) and 5-year survival (B) in TCGA datasets. The calibration curve of 1-year (C) and 5-year survival (D) in GSE65904 datasets. The 45º dashed line represented a perfect uniformity between nomogram-predicted and real possibilities.

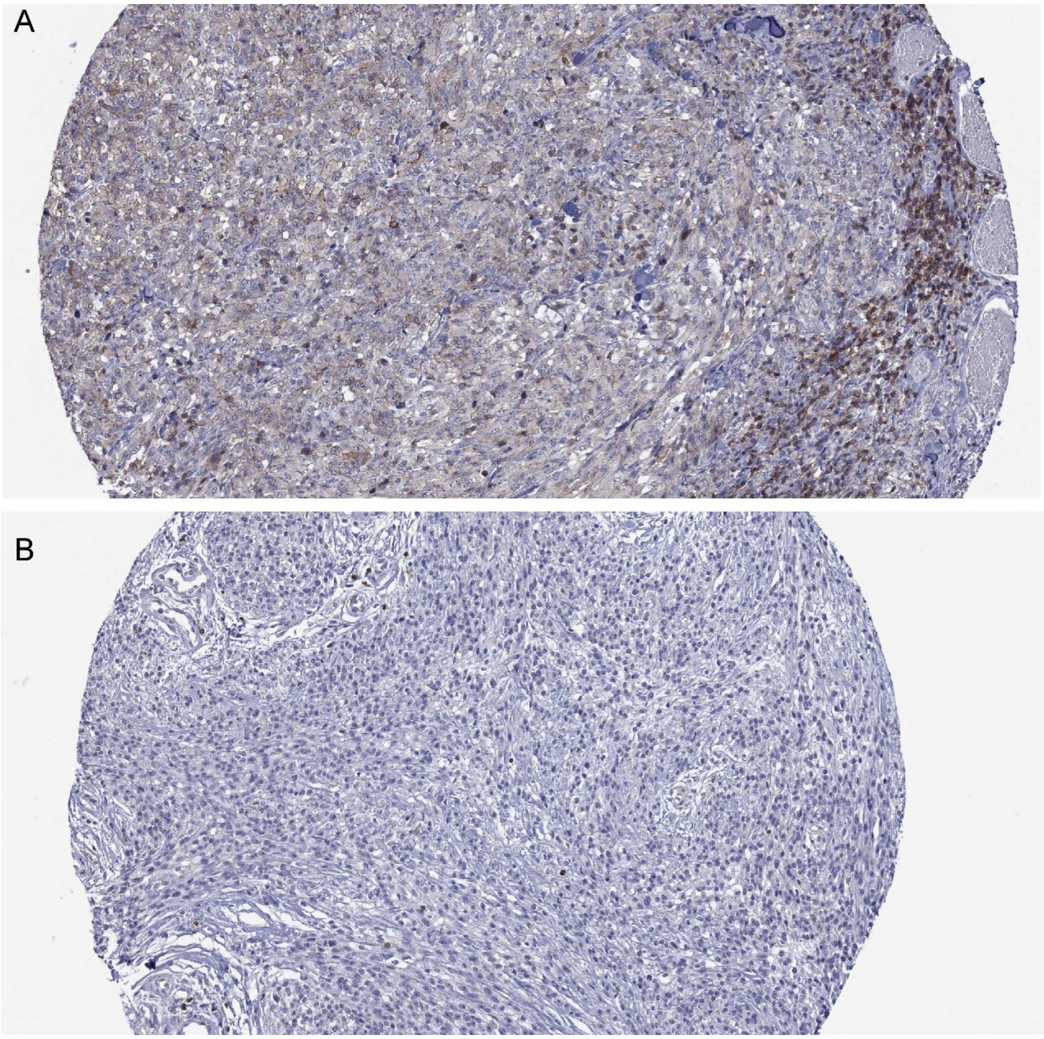

Figure S8. Different SIT1 protein expression in SKCM tumor cells. Positive (A) and negtive (B) expression of SIT1 protein in SKCM tumor cells.

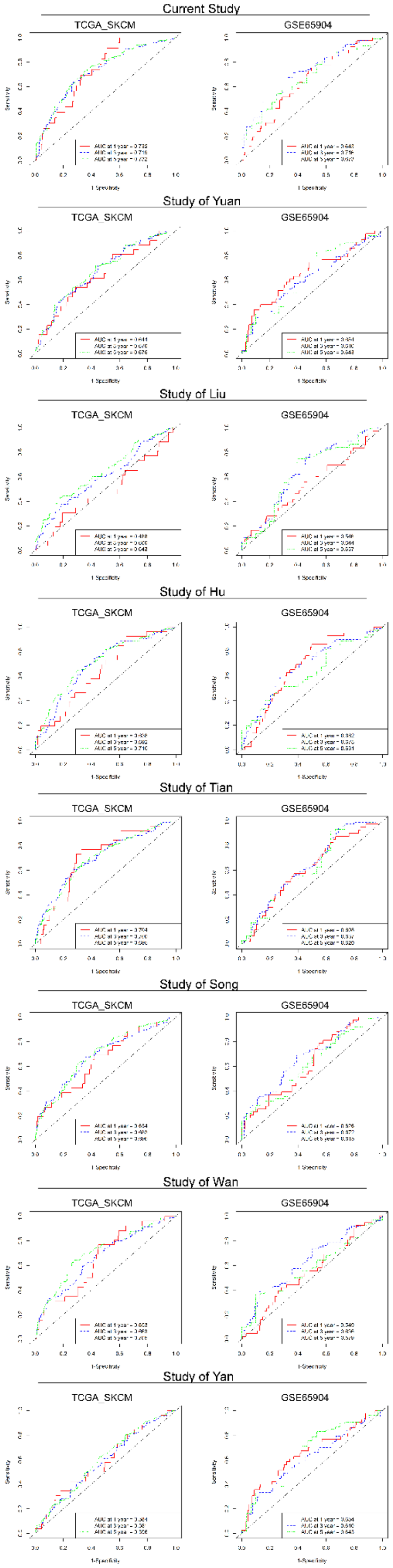

Figure S9. Comparisons of the predictivity between this study and other studies. (A) Predictivity of our risk score in melanoma patients' 1-year, 3-year and 5-year survival from dataset TCGA_SKCM and GSE65904. (B) Predictivity of Yuan's study in melanoma patients' 1-year, 3-year and 5-year survival from dataset TCGA_SKCM and GSE65904. (C) Predictivity of Liu's study in

melanoma patients’ 1-year, 3-year and 5-year survival from dataset TCGA_SKCM and GSE65904. (D) Predictivity of Hu’s study in melanoma patients’ 1-year, 3-year and 5-year survival from dataset TCGA_SKCM and GSE65904. (E) Predictivity of Tian’s study in melanoma patients’ 1-year, 3-year and 5-year survival from dataset TCGA_SKCM and GSE65904. (F) Predictivity of Song’s study in melanoma patients’ 1-year, 3-year and 5-year survival from dataset TCGA_SKCM and GSE65904. (G) Predictivity of Wan’s study in melanoma patients’ 1-year, 3-year and 5-year survival from dataset TCGA_SKCM and GSE65904. (H) Predictivity of Yan’s study in melanoma patients’ 1-year, 3-year and 5-year survival from dataset TCGA_SKCM and GSE65904.

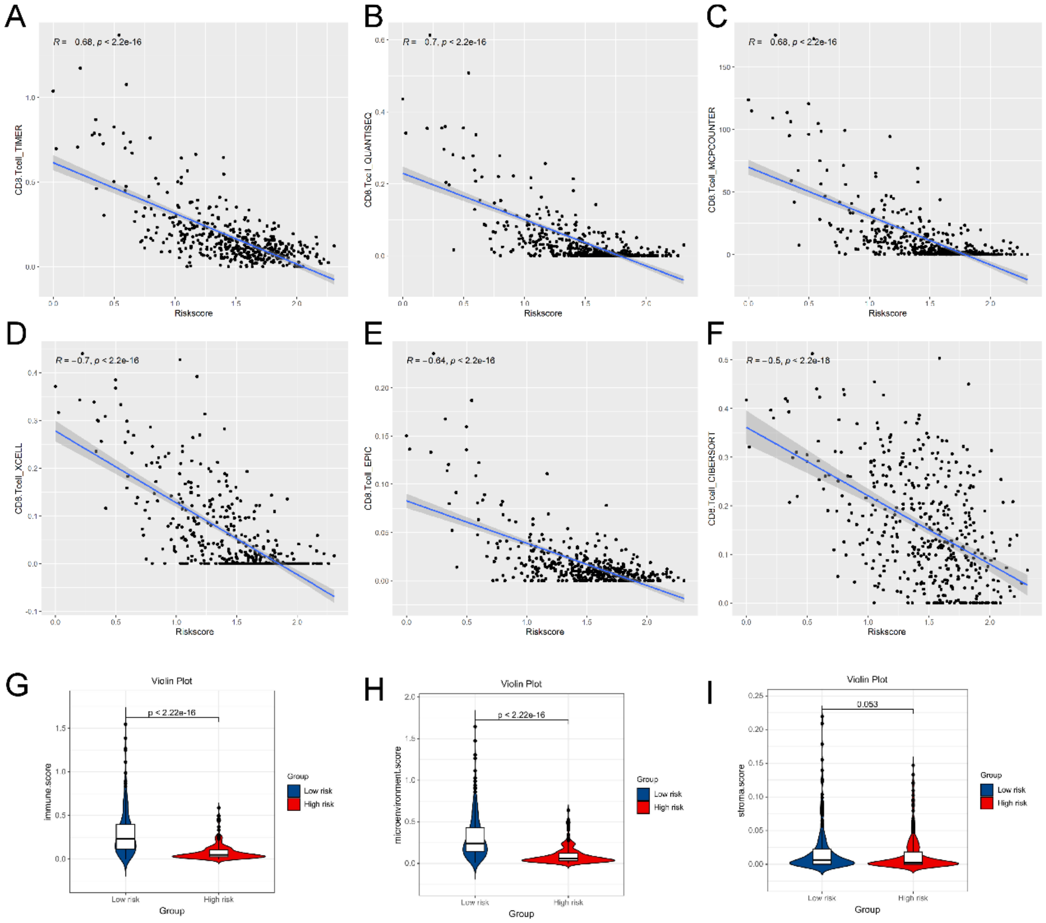

Figure S10. Relation between risk score and immune cell infiltration. (A-F) Correlation between risk score and the infiltrating number of CD8 T cells in melanoma patients from SKCM_TCGA dataset by analysis with TIMER (A), QUANTISEQ (B), MCPCOUNTER (C), XCELL (D), EPIC (E) and CIBESORT (F). ESTIMATE analysis of immune score (G) and microenvironment score (H) shows a significant difference between high risk and low risk melanoma patients. (I) Stromal score shows a boardline significant difference between high risk and low risk melanoma patients.

Table S1. Correlation analysis between SIT1 and markers of immune cells in SKCM of TIMER and GEPIA.

		TIMER				GEPIA	
		None		Purity		Tumour	
Description	Gene markers	Cor	*P* Value	Cor	*P* Value	Cor	*P* Value
CD8+ T cell	CD8A	0.923	***	0.880	***	0.60	***
	CD8B	0.933	***	0.891	***	0.47	***
T cell (general)	CD3D	0.979	***	0.963	***	0.63	***
	CD3E	0.984	***	0.972	***	0.67	***
	CD2	0.973	***	0.952	***	0.68	***
B cell	CD19	0.775	***	0.695	***	0.24	***
	CD79A	0.823	***	0.742	***	0.17	***
Monocyte	CD86	0.815	***	0.703	***	0.36	***
	CD115(CSF1R)	0.745	***	0.616	***	0.51	***
TAM	CCL2	0.579	***	0.384	***	0.16	***

	CD68	0.491	***	0.300	***	0.26	***
	IL10	0.596	***	0.429	***	0.21	***
M1 Macrophage	INOS(NOS2)	0.021	6.55E-01	0.016	7.37E-01	0.08	7.20E-02
	IRF5	0.667	***	0.483	***	0.06	1.40E-01
	COX2(PTGS2)	-0.036	4.33E-01	-0.167	***	0.09	3.40E-02
M2 Macrophage	CD163	0.597	***	0.443	***	0.25	***
	VSIG4	0.590	***	0.451	***	0.26	***
	MS4A4A	0.687	***	0.550	***	0.41	***
Neutrophils	CD66b (CEACAM8)	-0.071	1.23E-01	-0.052	2.64E-01	0.02	5.90E-01
	CD11b (ITGAM)	0.638	***	0.515	***	0.44	***
	CCR7	0.861	***	0.791	***	0.34	***
Natural killer cell	KIR2DL1	0.430	***	0.299	***	0.21	***
	KIR2DL3	0.611	***	0.456	***	0.19	***
	KIR2DL4	0.763	***	0.659	***	0.21	***
	KIR3DL1	0.601	***	0.470	***	0.26	***
	KIR3DL2	0.697	***	0.561	***	0.17	***
	KIR3DL3	0.221	***	0.177	***	0.04	3.60E-01
	KIR2DS4	0.497	***	0.392	***	0.24	***
Dendritic cell	HLA-DPB1	0.874	***	0.784	***	0.52	***
	HLA-DQB1	0.808	***	0.684	***	0.21	***
	HLA-DRA	0.867	***	0.773	***	0.46	***
	HLA-DPA1	0.850	***	0.766	***	0.40	***
	BDCA-1(CD1C)	0.665	***	0.499	***	0.46	***
	BDCA-4(NRP1)	0.259	***	0.129	5.66E-03	0.33	***
	CD11c (ITGAX)	0.628	***	0.435	***	0.52	***
Th1	T-bet (TBX21)	0.936	***	0.895	***	0.57	***
	STAT4	0.795	***	0.693	***	0.40	***
	STAT1	0.643	***	0.567	***	0.26	***
	IFN-γ (IFNG)	0.826	***	0.739	***	0.24	***
	TNF-α (TNF)	0.718	***	0.564	***	0.10	1.40E-02
Th2	GATA3	0.803	***	0.644	***	-0.15	***
	STAT6	0.051	2.70E-01	0.112	1.68E-02	0.04	4.10E-01
	STAT5A	0.279	***	0.367	***	0.22	***
	IL13	0.223	***	0.142	2.36E-03	0.15	***
Tfh	BCL6	0.267	***	0.177	***	-0.12	6.10E-03
	IL21	0.579	***	0.473	***	0.10	2.00E-02
Th17	STAT3	0.203	***	0.185	***	0.22	***
	IL17A	-0.056	2.26E-01	-0.151	1.24E-03	0.01	8.60E-01
Treg	FOXP3	0.851	***	0.752	***	0.26	***
	CCR8	0.776	***	0.679	***	0.29	***
	STAT5B	0.250	***	0.359	***	0.19	***
	TGFβ (TGFB1)	0.415	***	0.241	***	0.29	***
T cell exhaustion	PD-1 (PDCD1)	0.944	***	0.909	***	0.44	***
	CTLA4	0.578	***	0.411	***	0.49	***
	LAG3	0.896	***	0.839	***	0.27	***
	TIM-3 (HAVCR2)	0.817	***	0.701	***	0.41	***
	GZMB	0.867	***	0.769	***	0.44	***

TAM: tumour-associated macrophage; Th: T helper cell; Tfh: Follicular helper T cell; Treg: regulatory T cell; Cor: R value of Spearman's correlation; none: correlation without adjustment; purity: correlation adjusted by purity. ****P* < 0.001.

Table S2. Functions of the genes included in the prognostic signatures.

Gene symbol	Name	Function
CD80	T-Lymphocyte Activation Antigen CD80	Involved in the costimulatory signal essential for T-lymphocyte activation.
ICOSLG	Inducible T Cell Costimulator Ligand	Acts as a costimulatory signal for T/B-cell proliferation and cytokine secretion.

IL2RA	Interleukin 2 receptor subunit alpha	Regulation of immune Tolerance by controlling regulatory T cells (TREGs) activity.
KLRK1	Killer Cell Lectin Like Receptor K1	Provides both stimulatory and costimulatory innate immune responses on activated killer (NK) cells, leading to cytotoxic activity.
TMIGD2	Transmembrane And Immunoglobulin Domain Containing 2	Enhances T-cell proliferation and cytokine production via an AKT-dependent signaling cascade.
TNFRSF14	TNF Receptor Superfamily Member 14	Functions in signal transduction pathways that activate inflammatory and inhibitory T-cell immune response.
TNFRSF4	TNF Receptor Superfamily Member 4	Is a costimulatory molecule implicated in long-term T-cell immunity.
TNFSF15	TNF superfamily member 15	Promotes activation of caspases and apoptosis.
TNFSF4	TNF superfamily member 4	Cytokine that co-stimulates T cell proliferation and cytokine production.
ADORA2A	Adenosine receptor A2a	Plays an important role in many biological functions, such as cardiac rhythm and circulation, cerebral and renal blood flow, immune function, pain regulation, and sleep.
CD274	Programmed Cell Death 1 Ligand 1	Plays a critical role in induction and maintenance of immune tolerance to self.
IDO1	Indoleamine 2,3-Dioxygenase 1	Involved in the peripheral immune tolerance, contributing to maintain homeostasis by preventing autoimmunity or immunopathology that would result from uncontrolled and overreacting immune responses.
NECTIN2	Nectin Cell Adhesion Molecule 2	Modulator of T cell signaling.

Table S3. Comparison of clinicopathological features between the low- and high-risk subgroups of the TCGA_SKCM dataset.

Clinicopathological variable		Low-risk	High-risk	Chi-square test *P*-value	Spearman correlation coefficient	*P*-value
Age	<=60 y	134	112	**0.047**	-0.098	**0.037**
	>60 y	91	113			
Breslow	< 2cm	83	51	**<0.0001**	0.246	**<0.0001**
	>= 2cm	78	134			
clark	I-III	60	37	**0.002**	0.185	**0.001**
	IV-V	89	123			
M	M0	201	200	0.685	0.03	0.536
	M1	10	13			
N	N0	112	111	0.981	0.006	0.901
	N+	89	86			
T	T0-T2	90	56	**<0.0001**	0.224	**<0.0001**
	T3-T4	90	143			
BRAF mutation	No	99	125	**0.0142**	-0.116	**0.014**
	Yes	126	100			

Gender	female	94	77	0.12	0.078	0.099
	male	131	148			
Stage	0-II	107	118	0.614	-0.003	0.584
	III-IV	96	94			
Status	Alive	136	95	**0.0002**	0.182	**<0.0001**
	Dead	89	130			

Table S4. Comparison of clinicopathological features between the low- and high-risk subgroups of the GSE69540 dataset.

Clinicopathological variable		Low-risk	High-risk	Chi-square test *P*-value	Spearman correlation coefficient	*P*-value
Age	<=60 y	45	38	0.366	0.072	0.298
	>60 y	59	67			
Gender	female	46	40	0.483	0.058	0.402
	male	59	65			
Stage	0-II	9	17	0.134	-0.120	0.089
	III-IV	93	84			
Status	Alive	61	47	0.073	0.133	0.054
	Dead	44	58			
